# Core-Shell Structure Strategy to Prepare Super-tough Poly(lactic acid) Composites with Balanced Stiffness and Toughness

**DOI:** 10.1021/polymscitech.6c00012

**Published:** 2026-04-14

**Authors:** Wei Bao, Xiaodong Wang, Lei Li, Jing Jin, Yanxiong Pan, Hongwen Liang, Xiangling Ji, Wei Jiang

**Affiliations:** † University of Science and Technology of China, Hefei 230026, People’s Republic of China; ‡ State Key Laboratory of Polymer Science and Technology, Changchun Institute of Applied Chemistry, 58277 Chinese Academy of Sciences, Changchun 130022, People’s Republic of China; § State Key Laboratory of New Textile Materials and Advanced Processing Technologies and Key Laboratory of Textile Fiber and Products of Ministry of Education, College of Materials Science and Engineering, 216798 Wuhan Textile University, Wuhan 430200, People’s Republic of China; ∥ Hunan Petrochemical Co Ltd, Sinopec, Yueyang 414014, People’s Republic of China

**Keywords:** Poly(lactic acid), Balanced stiffness and toughness, Core-shell structure, Polyether block amide, Melt blending

## Abstract

Super-tough poly­(lactic acid) (PLA) composites with an excellent balance between stiffness and toughness were successfully developed by incorporating core-shell particles comprising a rigid silica (SiO_2_) core and an elastomeric poly­(ether-*block*-amide) grafted with a glycidyl methacrylate (PEBA-GMA) shell. Morphological observations confirmed the formation of a well-defined core-shell structure within the PLA matrix. The effects of SiO_2_ particle size (100–800 nm) and core-shell mass ratio (1:3 to 3:1) on mechanical properties were systematically investigated. The results indicate that smaller SiO_2_ core particles (100 nm) led to severe agglomeration and poor dispersion, resulting in ineffective toughening. The optimal performance was achieved with PLA composites containing 500 nm SiO_2_ at a core-shell ratio of 1:3 and a total modifier content of 20 wt %, which exhibited a notched Izod impact strength of 70.6 kJ/m^2^—a significant enhancement over binary PLA/PEBA-GMA composites. Furthermore, this composite maintained ductile fracture behavior even at a high SiO_2_ content, with an impact strength of 33.9 kJ/m^2^ at a SiO_2_:PEBA-GMA core-shell ratio of 3:1. The flexural modulus of this optimized composite was retained at 87% of that of pure PLA, demonstrating an excellent balance between stiffness and toughness. This work presents an effective strategy for developing high-performance PLA materials with tailored mechanical properties, offering promising potential for advanced biodegradable applications.

## Graphical abstract

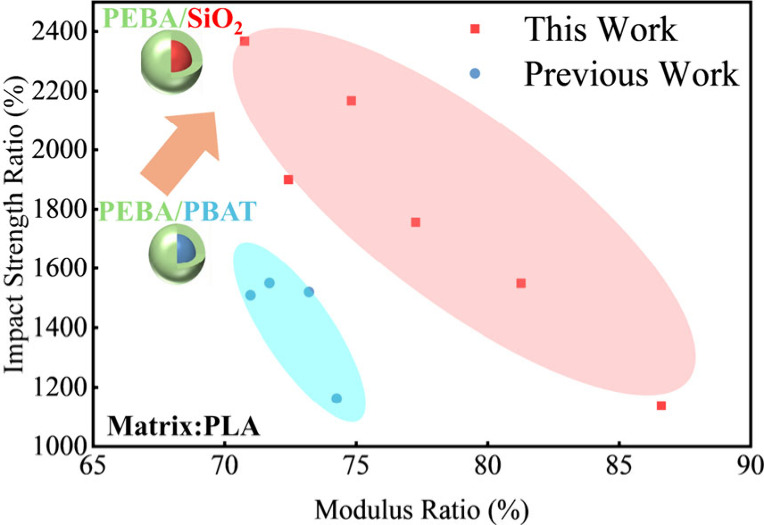

## 1 Introduction

The growing environmental burden caused by persistent petroleum-based plastics has intensified global efforts toward sustainable alternatives. Within this landscape, polylactic acid (PLA) has emerged as a leading bio-based and biodegradable polymer.^1–3^ PLA offers attractive properties, including biocompatibility, industrial composability, high tensile strength, rigidity, and compatibility with conventional processing techniques. Consequently, it finds extensive application in packaging, disposable products, and biomedical devices, positioning it as a key material in the bioplastics economy.^4,5^

Despite its many advantages, the widespread adoption of PLA in more demanding structural and durable applications is critically hampered by its inherent brittleness.^6–9^ Toughening methods for PLA are generally divided into chemical and physical approaches.^10–13^ The chemical approach involves synthesizing copolymers of PLA with other polymers to enhance its toughness.^14,15^ Although they are effective, these routes often require complex synthetic procedures and elevated cost and offer limited tunability for different application scenarios. In contrast, physical blending with elastomers or flexible polymers is more industrially attractive due to its simplicity and scalability.^16–24^ However, substantial toughening generally demands high elastomer loadings (≈20 wt %), which inevitably leads to pronounced reductions in tensile strength and stiffness.^17^ Therefore, overcoming this stiffness-toughness trade-off is crucial for expanding PLA into high-performance structural applications.

To address the inherent stiffness–toughness trade-off, core–shell particles comprising a soft, low-modulus shell and a rigid, high-modulus core have been proposed as an effective toughening strategy. Both experimental evidence and theoretical analyses have demonstrated that core–shell structures can markedly mitigate the modulus loss typically associated with elastomer toughening.^25–27^ Zheng *et al.* ^28^ successfully prepared polypropylene (PP)/ethylene-propylene rubber (EPR)/polyethylene (PE) blends with a core-shell structure, and found that the introduction of HDPE effectively improved the impact properties of the material and maintained a low loss of rigidity. Jiang *et al*.^25,29^ quantitatively calculated results show that compared with single-phase modified particles, the stiffness loss of core-shell rubber particle toughened polymer composites is smaller. At the same time, the results show that in order to obtain high impact polymer composites with low stiffness loss, the core modulus in core-shell particles should be as high as possible and the shell modulus should be as low as possible under the premise of ensuring the effectiveness of core-shell rubber particles.

In our previous work,^30^ we developed highly tough and biodegradable PLA blends featuring an in situ formed core–shell structure, where poly­(butylene adipate-*co*-terephthalate) (PBAT) acted as the core and polyesteramide (PBEA) served as the shell. Although the incorporation of PBAT effectively improved impact toughness, subsequent mechanical analysis revealed a critical limitation, i.e. the low modulus of PBAT (∼80 MPa) was insufficient to contribute meaningfully to the stiffness of the composites. Even though PBAT is considerably stiffer than the PBEA shell (<10 MPa), its modulus remains only about one-15th that of the PLA matrix (3–4 GPa). As a result, the stiffness of the PLA/PBEA/PBAT blends, while marginally higher than that of PLA toughened solely with elastomers, still fell far below that of neat PLA. This modulus mismatch highlights the need for a core material with substantially higher rigidity to achieve a truly balanced stiffness–toughness profile.

The quest for a suitable, biodegradable core material with a high modulus significantly exceeding that of PBAT (∼80 MPa) is currently a problem that needs to be addressed. Although several commercially available biodegradable polymers—such as poly­(butylene succinate) (PBS, ∼200 MPa)^31^ and poly­(3-hydroxybutyrate) (PHB, ∼1.5 GPa)^32^—possess higher stiffness, their moduli are still far below that of PLA (3–4 GPa). As a result, these polymers can provide only marginal improvements in composite rigidity and are unable to overcome the fundamental stiffness deficiency inherent in elastomer-toughened PLA systems.

To overcome this limitation and achieve a high-modulus core without compromising the essential biodegradability of the composite system, we propose a paradigm shift: employing rigid inorganic particles as the core material within the core-shell structure. Crucially, inorganic particles such as silica inherently exist in nature and do not compromise the biodegradable character of the overall material.

Among various inorganic particles, silica (SiO_2_) nanoparticles stand out as exceptionally promising candidates for this role. SiO_2_ is abundantly available, cost-effective, chemically inert, and possesses an extremely high Young’s modulus (∼70 GPa), far exceeding that of PLA and any biodegradable polymer.^33,34^ Owing to these properties, SiO_2_ has been widely employed in polymer nanocomposites, where it consistently contributes to enhanced stiffness, strength, dimensional stability, and barrier performance. Jiang *et al*.^33^ further demonstrated the effectiveness of SiO_2_-based core–shell particles, showing that encapsulating SiO_2_ with an elastomeric shell can produce polypropylene composites with simultaneously high impact strength (44.6 kJ/m^2^) and high flexural modulus (1644 MPa). These findings suggest that integrating SiO_2_ as a rigid core provides a promising pathway for achieving a favorable stiffness–toughness balance in polymer blends.

Building upon this successful strategy in conventional plastics and driven by the urgent need to overcome the rigidity limitations of biodegradable polymer cores in PLA toughening, this study adopts rigid SiO_2_ nanoparticles as the core material. We aim to design and fabricate PLA composites with a balanced stiffness-toughness profile and a core-shell structure while retaining essential biodegradability.

## 2 Experimental Section

### 2.1 Materials

The materials used in this study and their sources were listed below. Poly­(lactic acid) (PLA, grade 4032D) was supplied by NatureWorks LLC (USA), with a melt flow index of 4.13 g/10 min (190 °C, 2.16 kg). Surface-modified silicon dioxide (SiO_2_) nanoparticles (Commercial code: 0034) with five different average sizes (100, 200, 300, 500, and 800 nm) were provided by a Chinese supplier. The particles were functionalized with the silane coupling agent KH-550 at a grafting density of 1.57%. The poly­(ether block amide) (PEBA) elastomer, PEBAX® 25R33 SP01, was purchased from Arkema (France). Glycidyl methacrylate (GMA) and N-vinyl-2-pyrrolidinone (NVP) were procured from Macklin Biochemical Co., Ltd. Dicumyl peroxide (DCP) was obtained from Aladdin Biochemical Technology Co., Ltd.

### 2.2 Sample Preparation

The melt reaction grafting of the PEBA elastomer was performed with a Haake torque rheometer (model XSS-300, Shanghai, China). PEBA pellets were initially loaded and premixed at 180 °C for 2 min. Subsequently, dicumyl peroxide (DCP), glycidyl methacrylate (GMA), and N-vinyl-2-pyrrolidinone (NVP) were added, and the blending continued for a total mixing time of 5 min. The weight ratio of the components was fixed at PEBA : GMA : NVP : DCP = 100 : 3 : 3 : 0.3. The preparation and structural characterization of PEBA-GMA were detailed in our previous work,^30^ the grafting degree of GMA grafted onto PEBA was 1.5%.

The PLA composites were compounded as listed in Table S1 using the same rheometer at a temperature of 190 °C and a rotor speed of 80 rpm for 5 min. Specimens for mechanical testing (notched Izod impact, flexural, and tensile) were prepared using a plate vulcanizing machine (manufactured by Qingdao Yadong Rubber Machinery Co., Ltd., China). The compression molding process involved maintaining the material at 190 °C under a pressure of 10 MPa for 5 min, followed by cooling to room temperature under the same pressure for an additional 5 min.

### 2.3 Mechanical Properties

The notched Izod impact strength was measured according to Chinese Standard GB/T 1843-2008. The specimens had dimensions of 63.5 × 12.7 × 3.2 mm^3^ with a notch depth of 2.5 mm. Flexural tests were conducted following GB/T 9341-2008 on bar specimens measuring 80 × 10 × 4.0 mm^3^ at a cross-head speed of 2 mm/min. Tensile properties were evaluated based on GB/T 1040.1-2006 using dumbbell-shaped specimens (50 × 4.0 × 1.0 mm^3^) tested at a crosshead speed of 10 mm/min. Prior to testing, all specimens were conditioned at room temperature (23 ± 2 °C) for at least 24 h. Each reported mechanical property value represents the average of five successful tests. Impact tests were performed on an XJU-2.75 Izod impact tester (Chengde Testing Machine Factory, China). Both flexural and tensile tests were carried out on an Instron-1121 universal testing machine (UK).

### 2.4 Scanning Electron Microscopy (SEM)

The phase morphology of the PLA composites was observed using a Zeiss Gemini 300 scanning electron microscope operated at an accelerating voltage of 5.0 kV. To observe the dispersion of the core particles, the PLA composite samples were cryo-fractured after immersion in liquid nitrogen for 0.5 h. The fractured surfaces were then etched in n-butanol at 80 °C for 12 h to selectively dissolve and remove the PEBA-GMA shell phase. Subsequently, the etched and dried fracture surfaces were sputter-coated with a thin layer of gold prior to SEM observation.

### 2.5 Contact Angle Measurement

Interfacial tension values between PLA, PEBA-GMA, and SiO_2_ were determined indirectly via contact angle measurements, which were conducted using a DSA 100 contact angle goniometer (KRÜSS GmbH, Germany). All polymer samples were compression-molded into smooth sheets of approximately 1 mm thick for testing. Two probe liquids with different polarities, water and diiodomethane, were used for the measurements.

### 2.6 Differential Scanning Calorimetry (DSC)

The crystallinity of PLA blends was measured using differential scanning calorimetry (TA Instruments DSC Q2000, USA). Each sample was heated from 30°C to 200°C, held at 200°C for 3 min, and then cooled back to 30°C. The heating and cooling rates were both set at 10 °C/min. All measurements were conducted under a nitrogen purge. The crystallinity (Xc) was calculated using the eq 1, and *ΔHm* ^*0*^ is the theoretical melting enthalpy of 100% crystalline PLA (93.0 J/g).^35^ *ΔH*_*m*_ was the melting enthalpy of PLA in the PLA composite; Δ*H*_*cc*_ was the cold crystallization peak of PLA; *ω*_*PLA*_ was the mass fraction of PLA in PLA composites.

1 $$X_{C}\left(\%\right)=\frac{\Delta H_{m}-\Delta H_{cc}}{\omega_{PLA}\times \Delta H_{m}^{0}}\times 100\%$$

### 2.7 Rheological Test

Melt rheological properties were evaluated using a Discovery Hybrid Rheometer (DHR-1, TA Instruments, USA) equipped with a 25 mm parallel plate geometry. The samples were compression-molded into disks with a diameter of 25 mm and a thickness of 1 mm. The testing gap was set to 1 mm. Strain sweeps were conducted at 190 °C and 1 Hz for all samples to determine the linear viscoelastic region (LVR). Isothermal oscillatory tests at a frequency of 1 Hz and a strain amplitude of 1% were performed to test the melt stability of the PLA samples at 190 °C for up to 30 min. Dynamic frequency sweeps were performed from 100 to 0.1 rad/s at 190 °C under a nitrogen atmosphere to prevent thermal degradation.

## 3 Results and Discussion

### 3.1 Mechanical Properties

Figure 1 presents the notched Izod impact strength of the PLA/PEBA/SiO_2_ composites as a function of modifier content for different core-shell mass ratios and SiO_2_ core particle sizes. As expected, neat PLA displayed typical brittle behavior, with a very low impact strength of 3.0 kJ/m^2^, serving as the baseline for evaluating the toughening effectiveness of the designed core–shell structures.

**Figure 1:**
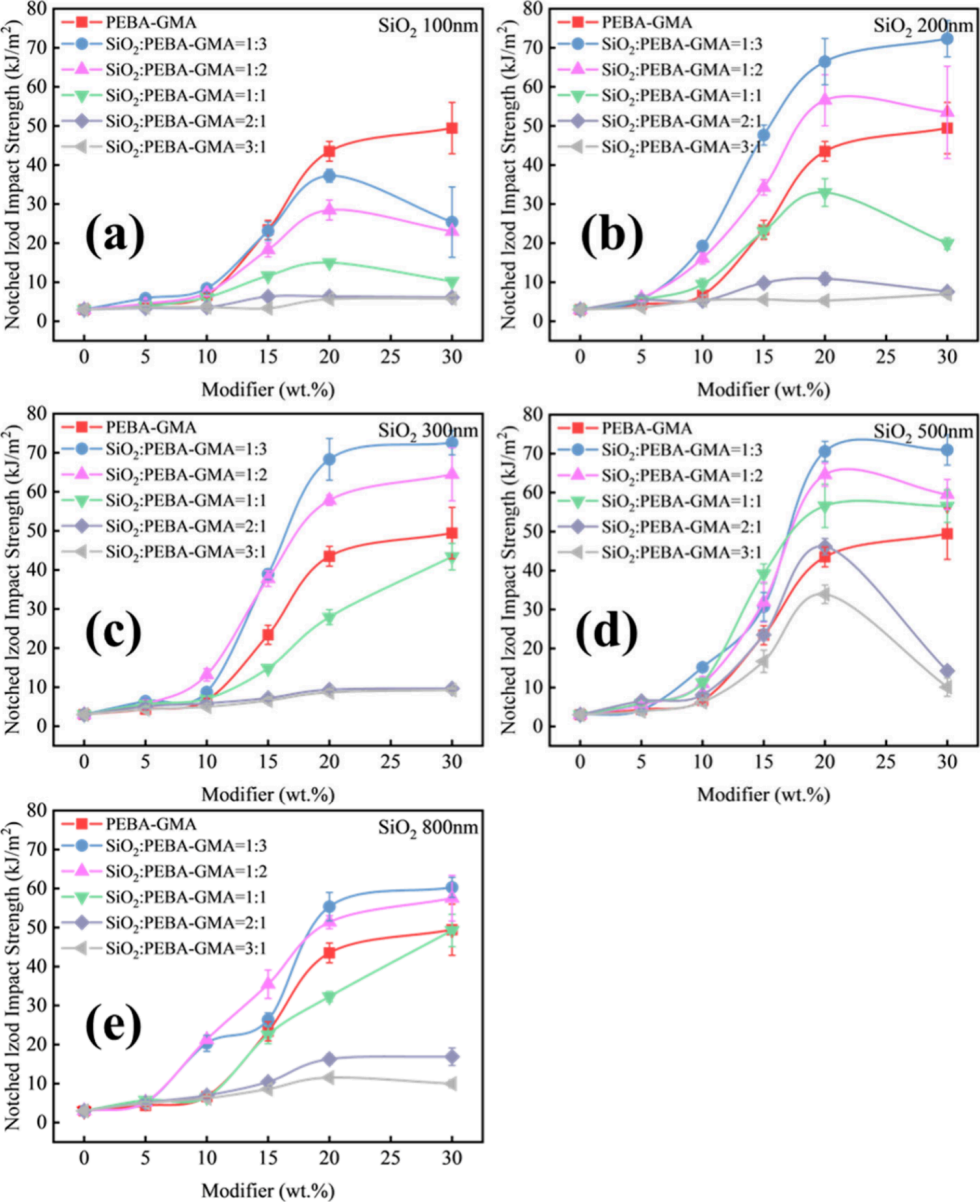
Notched Izod impact strength as a function of modifier content for PLA/PEBA-GMA/SiO_2_ composites with different core-shell mass ratios. The particle size of the core SiO_2_ is different: (a) 100 nm; (b) 200 nm; (c) 300 nm; (d) 500 nm; (e) 800 nm.

The toughening performance strongly depended on the SiO_2_ particle size. When 100 nm SiO_2_ was used (Figure 1a), the impact strength increased gradually with increasing modifier content, reaching a peak for 37.2 kJ/m^2^ at 20 wt %. When the addition of modifier increased to 30 wt %, it decreased to 25.3 kJ/m^2^. However, this value remained lower than that of the binary PLA/PEBA-GMA (80/20) blend (43.5 kJ/m^2^), demonstrating that the incorporation of 100 nm of SiO_2_ offered no synergistic toughening benefit. This inferior performance is attributed to the severe agglomeration tendency of ultrafine SiO_2_ nanoparticles, which impairs dispersion and restricts the activation of toughening mechanisms. This interpretation is consistent with the morphological observations discussed later.

In contrast, PLA composites containing larger SiO_2_ particles (200–800 nm) demonstrated significantly enhanced toughening performance. Specifically, the 500 nm system exhibited the strongest overall toughening capability across various core-shell ratios. For instance, at a SiO_2_:PEBA-GMA mass ratio of 1:2, the impact strength increased consistently with modifier content, reaching 64.6 kJ/m^2^ at 20 wt %. At a ratio of 1:3 of SiO_2_:PEBA-GMA, the impact strength peaked for 70.6 kJ/m^2^ at 20 wt %, substantially exceeding that of the blend modified with pure PEBA-GMA. This indicates that an optimal particle size provides a more effective stress concentration and triggers large-scale shear yielding in the matrix.

The evolution of modifier content from 0 to 30 wt % fully characterized the brittle-to-ductile transition of the materials. At a low loading level of 5 wt %, no significant enhancement in impact strength was observed across all systems, with values remaining below 10 kJ/m^2^. This was because the average distance between core-shell particles is too large to form an effective percolation network or trigger synergistic deformation of the matrix. When the content increases to 15-20 wt %, a critical interparticle distance is reached, fully activating multiple cavitation and shear yielding mechanisms, which results in a burst in impact strength. However, at an extremely high addition of 30 wt %, the toughening effect shows a saturation or declining trend for different core-shell mass ratio. Particularly for the high core content systems, excessive addition may lead to local aggregation, resulting in an increase in the particle size of the dispersed phase of core-shell particles, as shown in Figure S1, resulting in diminishing returns in toughening efficiency.

At a fixed core-shell mass ratio of 1:1, the impact strengths for PLA/PEBA/SiO_2_ (80/10/10) composites with 200, 300, 500, and 800 nm SiO_2_ were 32.9 kJ/m^2^, 27.9 kJ/m^2^, 56.5 kJ/m^2^, and 32.2 kJ/m^2^, respectively. The PLA composites with 500 nm SiO_2_ yielded the highest impact strength, indicating its superior ability to accommodate a higher SiO_2_ core content while maintaining ductile fracture behavior. Further investigation at other core-shell ratios revealed that at a SiO_2_:PEBA-GMA ratio of 2:1, the impact strength was 46.1 kJ/m^2^, comparable to that of the binary PLA/PEBA-GMA (80/20) blend. Even at a higher core-shell ratio of 3:1 of SiO_2_:PEBA-GMA, the impact strength remained at 33.9 kJ/m^2^, still indicative of ductile fracture, albeit reduced.

A balance between the stiffness and toughness is crucial for practical applications. Figure 2a shows the variation of the flexural modulus with modifier content for composites with 500 nm SiO_2_. The flexural modulus of all composites decreased with increasing modifier content. Notably, PLA composites with a higher core content in core-shell particles exhibited a relatively higher modulus. When the total modifier content was 20 wt % and the core-shell ratio was 3:1­(SiO_2_:PEBA-GMA), the flexural modulus of the PLA composite was 3400 MPa, retaining 87% of the modulus of pure PLA.

**Figure 2:**
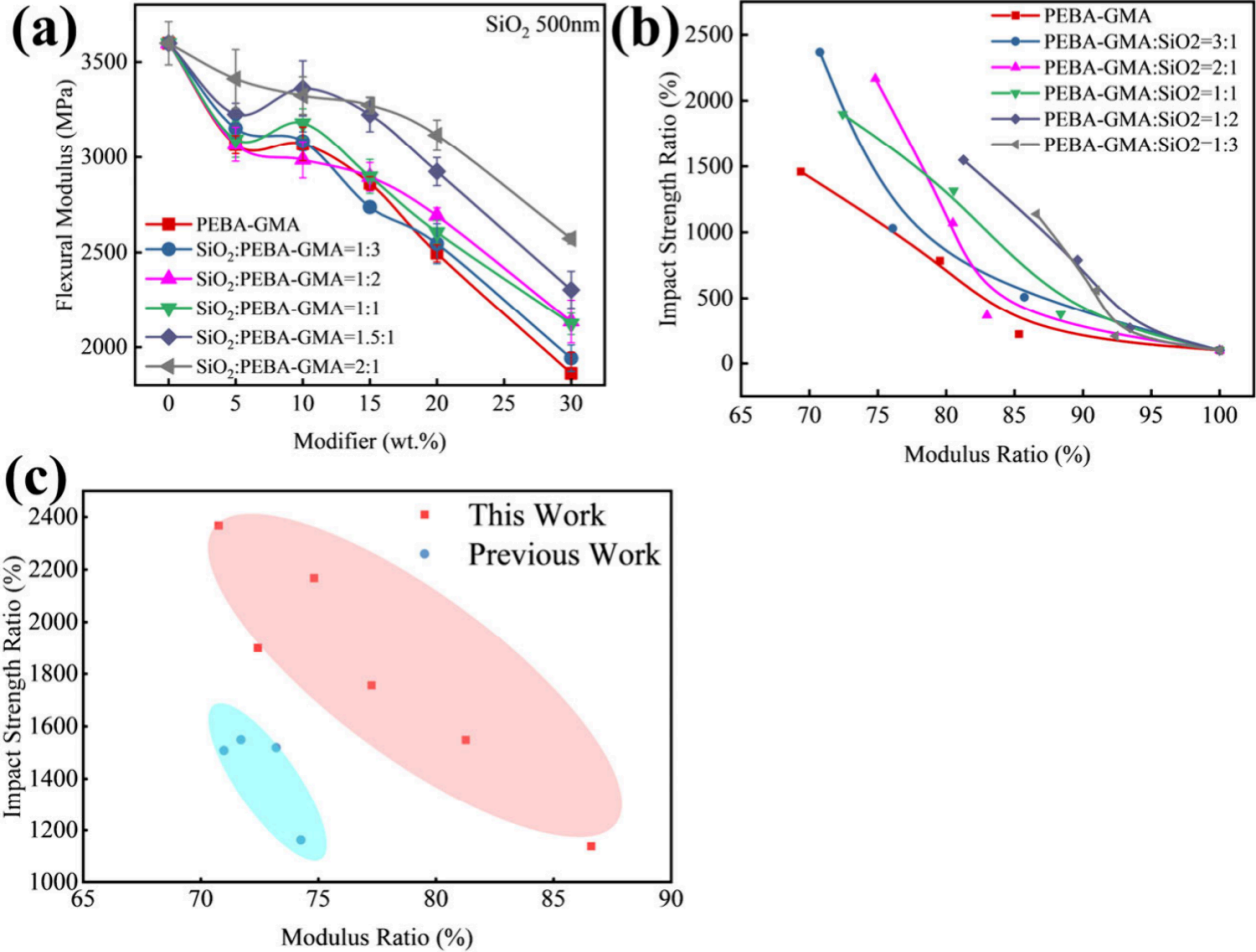
(a) Flexural modulus as a function of modifier content for PLA/PEBA/SiO_2_ composites with different core-shell mass ratios. The particle size of core SiO_2_ is 500 nm. (b) Impact strength ratio as a function of modulus ratio of PLA/PEBA-GMA/SiO_2_ composite to pure PLA. (c) Stiffness and toughness of PLA composites compared to previous works.^30^

Figure 2b presents the relationship between the normalized impact strength and normalized flexural modulus for the PLA/PEBA-GMA/SiO_2_ composites. Compositions positioned closer to the upper-right region of the plot exhibit a superior stiffness–toughness balance. The optimized formulation identified in this study achieves an impact strength nearly 11 times higher than that of neat PLA while retaining 87% of its original modulus. This performance highlights the exceptional ability of the SiO_2_–PEBA-GMA core–shell particles to produce synergistic enhancements in toughness with only minimal compromise in stiffness. As further illustrated in Figure 2c, the PLA/PEBA-GMA/SiO_2_ composites developed in this work exhibited a markedly superior stiffness–toughness balance compared with previously reported PLA-based toughened.^30^

In addition to impact performance, the tensile properties presented in Table 1 further confirm the balanced stiffness and toughness of the PLA composites. The system with a high SiO_2_ content (core-shell ratio of 3:1) retained the highest tensile modulus (1678.3 MPa). In contrast, PLA composites with another core-shell ratios (1:1 and 1:2) exhibited excellent elongation at break (>200%) and tensile toughness (>6000 MJ/m^3^), reflecting the significant enhancement in energy dissipation enabled by the core-shell structure. These results align well with the impact performance trends, confirming that the SiO_2_/PEBA-GMA core-shell particles not only promote plastic deformation for toughening but also preserve the load-bearing capacity, successfully addressing the stiffness-toughness trade-off in PLA composites.

**Table 1:** Tensile Mechanical Properties of PLA and PLA/PEBA-GMA/SiO_2_-500 nm Composites with Different Core-Shell Ratios^a^

Item	Tensile Modulus (MPa)	Tensile Strength (MPa)	Elongation at break (%)	Tensile toughness (MJ/m^3^)
PLA	2094.8±80.0	69.5±6.9	20.3±6.2	423.0±249.9
PLA/PEBA-GMA/SiO_2_(1:3)	1407.0±54.0	34.1±1.9	185.3±40.8	5044.0±1040.4
PLA/PEBA-GMA/SiO_2_(1:2)	1478.5±58.2	39.5±0.3	205.5±38.2	6195.3±1292.4
PLA/PEBA-GMA/SiO_2_(1:1)	1510.7±52.9	40.5±1.3	210.8±48.0	6386.1±1609.3
PLA/PEBA-GMA/SiO_2_(2:1)	1428.6±123.3	39.9±1.1	172.9±73.7	5733.1±1310.5
PLA/PEBA-GMA/SiO_2_(3:1)	1678.3±59.7	43.0±1.0	46.2±10.0	1472.3±357.2

a The addition of core-shell particles is 20 wt %.

### 3.2 Morphology Analysis

Figure 3 presents SEM images of the phase morphology for PLA/PEBA-GMA/SiO_2_ composites with different SiO_2_ core sizes and core-shell ratios after the PEBA shell was selectively etched away. Numerous cavities, from which the PEBA shell was removed, were observed within the PLA matrix with spherical SiO_2_ cores remaining inside them. Pronounced agglomeration of SiO_2_ particles was evident when the core size was 100 nm (Figures a1-a3). This agglomeration was detrimental to toughening as it impeded efficient stress transfer and energy dissipation, which correlated well with the inferior impact strength results shown in Figure 1a. This observation was consistent with literature reports where nanoparticle agglomeration at small sizes compromised mechanical performance.^34^ In contrast, PLA composites with larger SiO_2_ particles (200-800 nm) displayed a more uniform distribution of the SiO_2_ cores within the cavities, contributing to their superior toughening effect.

**Figure 3:**
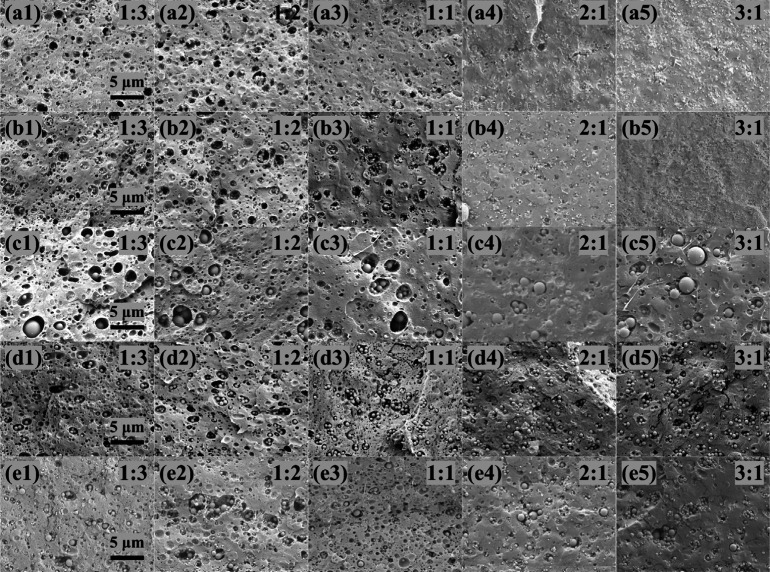
SEM images showing phase morphology for PLA/PEBA/SiO_2_ composites with different particle sizes of the core SiO_2_ and different SiO_2_–PEBA core-shell mass ratios: (a1-a3) 100 nm, (b1-b3) 200 nm, (c1-c3) 300 nm, (d1-d3) 500 nm, and (e1-e3) 800 nm. The PLA matrix was fixed at 80 wt %. The cryo-fracture surfaces were etched in n-butanol at 80 °C for 12 h to remove the PEBA.

Based on the etched cryo-fractured surfaces for the 100 and 500 nm SiO_2_ systems (both with a 1:1 core-shell ratio and 20 wt % total modifier content), the particle size of the core-shell particles was statistically analyzed using Nano-Measure software, As shown in Figure S2, the 100 nm SiO_2_ composite exhibits a broad size distribution with a number-average diameter of 0.74 μm, which was nearly eight times the primary particle size, and over 27% of the domains exceeding 1 μm. This confirmed severe agglomeration, which compromises toughening efficiency (impact strength of only 37.2 kJ/m^2^). In contrast, the 500 nm SiO_2_ composite displays a narrow size distribution centered around the nominal size (number-average diameter of 0.72 μm, only 1.4 times the primary size), with 90.4% of the domains between 0.4 and 1.0 μm.

This uniform dispersion and robust interfacial adhesion are fundamentally attributed to the chemical compatibility between the shell and the matrix. It is well recognized that a stable interface is essential for achieving effective toughening in polymer blends, as it enables efficient stress transfer and prevents premature failure.^36,37^ For instance, Shao *et al.* ^36^ demonstrated that anchoring carbon-based nanofillers at the PLA/poly­(butylene succinate) (PBS) interface via a Pickering-emulsion-assisted melt blending strategy significantly enhanced both mechanical strength and toughness while simultaneously improving triboelectric output. Similarly, Guo *et al*.^37^ showed that precisely localizing amphiphilic metal-organic frameworks (MOFs) at the PLA/PBS interface led to a remarkable 23-fold increase in tensile toughness, attributed to enhanced interfacial adhesion and chain entanglements.

As systematically demonstrated in our previous work using ^1^H NMR spectroscopy and selective extraction experiments,^30^ the epoxy groups of the GMA units grafted onto PEBA can undergo specific chemical reactions with the carboxyl or hydroxyl end groups of PLA chains during melt processing. This interfacial reaction effectively enhances the adhesion strength, thereby preventing particle agglomeration and ensuring efficient stress transfer from the PLA matrix to the elastomeric shell. Given that this study employs the same PEBA-GMA material and similar processing conditions, the same interfacial chemistry is expected to occur, ensuring strong interfacial adhesion and morphological stability.

Figure 4 shows TEM images of the PLA composites, where the core-shell structure was distinctly visible. As highlighted by the red circles, the light-colored regions corresponded to the shell material, while the spherical, dark-colored domains represented the SiO_2_ cores. This contrast provided direct visual evidence of the successful formation of SiO_2_–PEBA core-shell particles within the PLA matrix.

**Figure 4:**
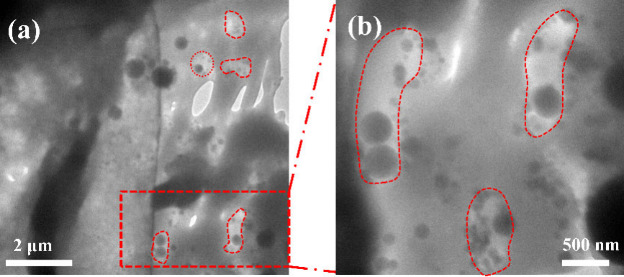
TEM images showing the phase morphology for PLA/PEBA-GMA/SiO_2_ composites. The particle size of the core SiO_2_ is 500 nm: (a) low-magnification image; (b) high-magnification image of the selected area in (a).

This well-defined core-shell morphology was crucial for achieving a balanced stiffness-toughness combination. The rigid SiO_2_ core, with its extremely high modulus (∼70–80 GPa), effectively enhanced the stiffness of composites, while the soft PEBA shell facilitated energy dissipation and toughening. This structural strategy aligned with reports of other ternary blend systems. For instance, Wu *et al*.^38^ demonstrated that a PBS/PBAT core–shell structure could significantly improve the impact strength of PLA blends while maintaining stiffness, attributing the enhancement to optimized particle size and interfacial compatibility. Similarly, Wang *et al*.^26^ reported that encapsulating rigid PA11 particles with an EGMA elastomer shell led to a remarkable increase in notched impact strength with minimal loss of rigidity, underscoring the importance of core–shell architectures in balancing mechanical properties.

### 3.3 Contact Angle Measurements and Interfacial Tension

The equilibrium morphology in ternary polymer blends is governed by interfacial interactions, which can be predicted using the spreading coefficient (λ) theory.^26,38^ According to the Harkins equation (eq 2) reformulated by Hobbs *et al*.^39^ for a system with a continuous matrix (component 1) and two dispersed minor phases (components 2 and 3), the spreading coefficient λ_23_ determines the resulting morphology:

2 $$\lambda_{23}=\gamma_{13}-\gamma_{12}-\gamma_{23}$$

where *γ*_12_, *γ*_13_ and *γ*_23_ represent the interfacial tensions between the respective components, as denoted by the subscripts. If *λ*_23_ is positive, component 3 will spontaneously be encapsulated by component 2, leading to the formation of a core (component 3)-shell (component 2) structure.

The interfacial tension *γ*_12_ between components 1 and 2 can be calculated using eq 3:

3 $$\gamma_{12}=\gamma_{1}+\gamma_{2}-4\times \left(\frac{\gamma^{d1}\gamma^{d2}}{\gamma^{d1}+\gamma^{d2}}+\frac{\gamma^{p1}\gamma^{p2}}{\gamma^{p1}+\gamma^{p2}}\right)$$

where *γ*_1_ and *γ*_2_ are the surface tensions of components 1 and 2, respectively; *γ* ^*d*^ and *γ* ^*p*^ denote the dispersive and polar components of the surface tension. The interfacial tensions γ_13_ and γ_23_ can be similarly calculated.

The surface tension (*γ*_1_) between a solid polymer and a liquid can be determined by solving the mean value of eq 4:

4 $$\gamma_{l}\left(1+\cos\theta \right)=4\left(\frac{\gamma_{l}^{d}\gamma_{s}^{d}}{\gamma_{l}^{d}+\gamma_{s}^{d}}+\frac{\gamma_{l}^{p}\gamma_{s}^{p}}{\gamma_{l}^{p}+\gamma_{s}^{p}}\right)$$

Here, the subscripts ″s″ and ″l″ refer to the solid and liquid, respectively. We used one polar liquid (water) and one dispersive liquid (diiodomethane) for this purpose. The dispersive and polar components of the surface free energy for water and diiodomethane are *γ*_*H*_2_ *O*_^*d*^= 22.1 mN/m,, *γ*_*H*_2_ *O*_^*p*^= 50.7 mN/m, *γ*_*CH*_2_ *I*_2__^*d*^= 44.1 mN/m, and *γ*_*CH*_2_ *I*_2__^*p*^= 6.7 mN/m respectively.^40^

The measured contact angles and the calculated surface tensions for PLA (matrix, component 1), PEBA-GMA (shell, component 2), and SiO_2_ (core, component 3) were summarized in Table 2. The corresponding interfacial tensions between each polymer pair, calculated using eq 3, were listed in Table 3.

**Table 2:** Contact Angle and Surface Tension for PLA, PEBA-GMA and SiO_2_-300 nm Components

	Contact angle (°)		Surface tension (mN/m)		
Sample	Water	Diiodomethane	Dispersion term (*γ* ^*d*^)	Polar term (*γ* ^*p*^)	Total (γ)
PLA	71.8	42.1	29.1	14.6	43.7
PEBA-GMA	78.5	30.5	35.5	9.8	45.3
SiO_2_-300 nm	103	62.6	27.9	1.8	29.7

**Table 3:** Interfacial Tension of Polymer Pairs and the Spreading Coefficient

Polymer pair	Interfacial tension (mN/m)
PLA/PEBA-GMA	*γ*_12_ = 1.5
PLA/SiO_2_	*γ*_13_ = 9.9
PEBA-GMA/SiO_2_	*γ*_23_ = 6.4

Based on the interfacial tensions in Table 2, the spreading coefficient *λ*_*23*_ (SiO_2_ covered by PEBA-GMA) was calculated to be 2.0 mN/m, which was positive. Conversely, the *λ*_*32*_ (PEBA-GMA covered by SiO_2_) was -5.0 mN/m, which was negative. This result provided a thermodynamic rationale for the observed morphology: the positive λ_23_ confirmed that SiO_2_ was energetically favored to be encapsulated by PEBA-GMA, forming the desired core-shell structure with SiO_2_ as the core and PEBA-GMA as the shell. This theoretical prediction was in excellent agreement with the direct morphological evidence obtained from SEM and TEM observations (Figures 3 and 4).

### 3.4 Rheological Properties

Melt rheology measurements were performed to evaluate the viscoelastic network structure and the dispersion state of the core-shell particles in the PLA matrix. Prior to frequency sweep measurements, strain sweep experiments confirmed that all samples exhibited a well-defined LVR up to at least 2% strain, as shown in Figure S3 (Supporting Information). Therefore, the strain used for subsequent frequency sweep measurements is 1%. Thermal stability demonstrated that neat PLA exhibited a gradual decrease in *G′* of approximately 13.0% over 30 min, while the PLA/PEBA-GMA/SiO_2_ composite with a 1:1 core-shell ratio showed a slightly lower decrease of 10.5%, as presented in Figure S4 (Supporting Information). All samples were tested under identical conditions, and the composites demonstrate thermal stability comparable to or better than that of neat PLA.

As shown in Figure 5a, neat PLA exhibited a typical linear viscoelastic behavior with a slope of approximately 0.9 in the low-frequency region, which is similar to that reported in the literature.^41^ Theoretically, a homopolymer melt should exhibit a terminal slope of 2. However, the observed deviation was attributed to the high molecular weight (Mw = 200 000 g/mol) and polydispersity of the commercial PLA matrix.^19^ The long relaxation times of the high-molecular-weight chains prevented the material from reaching the fully relaxed terminal zone within the tested frequency range (>0.1 rad/s). Nevertheless, this behavior was distinctly liquid-like compared to the PLA modified composites.

**Figure 5:**
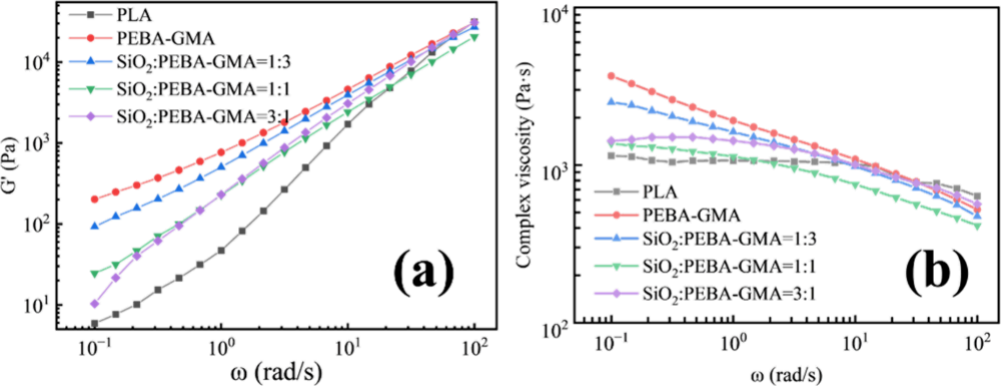
Rheological properties of PLA/PEBA-GMA/SiO_2_ composites with different SiO_2_:PEBA-GMA mass ratios as a function of frequency, (a) Storage modulus (*G’*), and (b) complex viscosity (η*). The PLA is fixed at 80 wt %, and the SiO_2_ particle size is 500 nm.

The incorporation of core-shell particles significantly altered this relaxation behavior. The PLA composites with a PEBA-GMA-rich shell (core-shell ratio of 1:3) displayed a much lower slope and the highest storage modulus (92.4 Pa at 0.1 rad/s). This flattening of the *G’* curve was indicative of the formation of a physical network formed by the entangled elastomeric shells, which restricted the long-range motion of PLA chains.

Increasing the rigid SiO_2_ content (core-shell ratio = 3:1) resulted in a restoration of the liquid-like behavior. The storage modulus of the 3:1 system dropped to 10.3 Pa at 0.1 rad/s and its low-frequency slope increased to approximately 1.3. This slope was notably steeper than that of the neat PLA, providing evidence that the rigid SiO_2_ cores effectively diluted and disrupted the elastomeric entanglement network. In the absence of sufficient elastomeric shell coverage, the particles behaved as isolated fillers that failed to restrict chain relaxation, resulting in a significant decrease in melt elasticity.

A similar trend was observed for the complex viscosity (Figure 5b). The introduction of the elastomeric PEBA-GMA phase significantly increased the complex viscosity in the low-frequency region compared to neat PLA, which typically hindered melt flow. However, interestingly, with the incorporation and increasing content of SiO_2_ cores, the complex viscosity exhibited a markedly decreasing trend. This indicated that the addition of SiO_2_ helped mitigate the excessive viscosity buildup caused by the rubber phase. This reduction in viscosity at a high SiO_2_ content implied that the core-shell particles could be incorporated without severely compromising the flowability of the matrix, which was highly beneficial for melt processing.

### 3.5 Thermal Properties

Differential scanning calorimetry (DSC) was employed to investigate the thermal properties and crystallinity of PLA composites. This analysis is crucial because the crystallinity of PLA is a well-established factor that significantly influences its toughness.^35,42^ For instance, Oyama^42^ demonstrated that a substantial increase in PLA crystallinity (up to 40%) induced by annealing was necessary to achieve a high impact strength of 72.0 kJ/m^2^ in PLA toughened with an E-MA-GMA elastomer. This underscored the importance of evaluating crystallinity’s contribution to toughness.

Figure 6 displays the first heating scans of neat PLA and various PLA/PEBA-GMA/SiO_2_ composites. All curves exhibited similar thermal transitions: an endothermic step change corresponding to the glass transition of PLA (*T*_*g,PLA*_=62.5 °C), a cold crystallization exotherm (*T*_*cc,PLA*_ = 102.6 °C), and a melting endotherm (*T*_*m,PLA*_=168.1 °C), as annotated for neat PLA. The enthalpy values and the calculated degree of crystallinity (*X*_*c*_) for neat PLA and its PLA composites were summarized in Table 4. The data indicated that the incorporation of modifiers generally reduced the cold crystallization temperature (*T*_*cc*_) and increased the crystallinity (*X*_*c*_) of PLA to some extent, suggesting a slight nucleating effect. However, the central finding was that the crystallinity of all PLA composites fall within a relatively narrow range (15.7% to 20.1%), despite the drastic variations in their impact strength (from ∼28 to over 70 kJ/m^2^, as shown in Figure 1). This clear discrepancy demonstrated that the dramatic differences in toughness observed in this study were not primarily governed by changes in crystallinity. Instead, the toughening mechanism must be dominantly associated with the unique phase morphology of the core-shell structure, which effectively triggered energy dissipation processes through cavitation and shear yielding.

**Figure 6:**
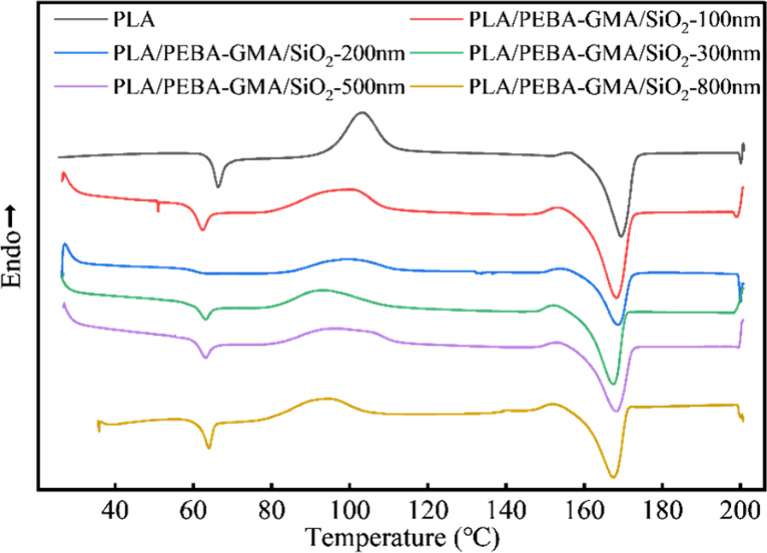
DSC curves during the first heating scan for neat PLA and PLA composites (the PLA is fixed at 80 wt %).

**Table 4:** Values of Enthalpy and Crystallinity for Neat PLA and PLA Composites with Different Modifiers^a^

Samples	*T*_*g*_ (°C)	*T*_*m*_ (°C)	*ΔH*_*m*_ (J/g)	*T*_*cc*_ (°C)	*ΔH*_*cc*_ (J/g)	*X*_*c*_ (%)
PLA	62.5	168.1	34.9	102.6	26.1	11.7
PLA/PEBA-GMA/SiO_2_-100 nm	60.5	168.2	36.9	100.1	25.2	15.7
PLA/PEBA-GMA/SiO_2_-200 nm	62.5	169.4	28.4	97.3	16.5	16.0
PLA/PEBA-GMA/SiO_2_-300 nm	61.8	167.3	31.1	93.3	17.4	18.4
PLA/PEBA-GMA/SiO_2_-500 nm	61.5	168.2	29.7	96.2	17.7	16.0
PLA/PEBA-GMA/SiO_2_-800 nm	62.2	167.8	26.9	93.6	11.9	20.1

a PLA is fixed at 80 wt %.

### 3.6 Toughening Mechanism

Based on the detailed analysis of the impact-fractured surfaces examined at different locations—from the notched region to the central and hinge areas—a progressive deformation mechanism mediated by the SiO_2_–PEBA-GMA core–shell particles can be proposed, as shown in Figure 7. The fracture process demonstrated a clear brittle-to-ductile transition, which follows the cavitation-induced shear yielding theory.^43^

**Figure 7:**
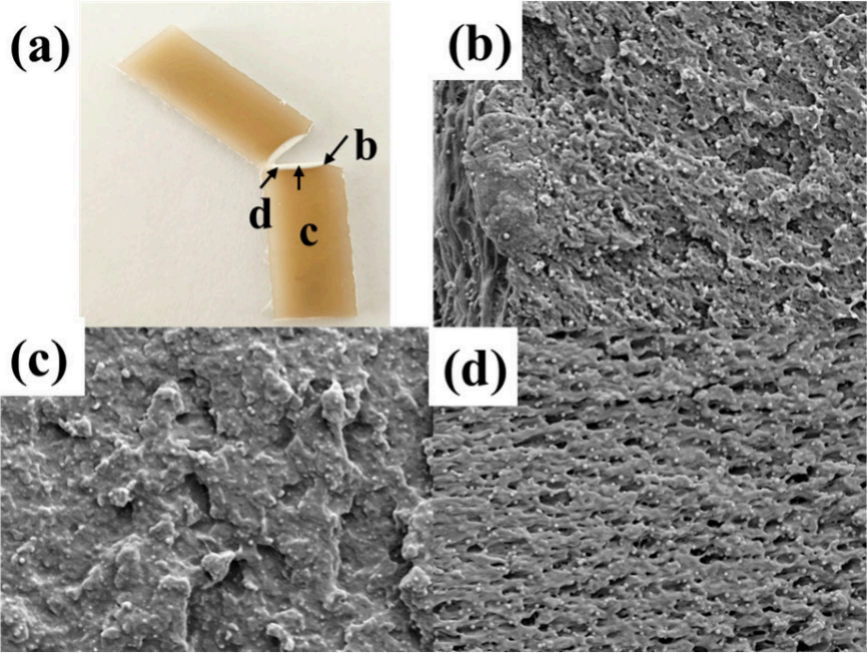
(a) Photograph of PLA/PEBA-GMA/SiO_2_ composite specimen after impacting test. (b-d) SEM images showing phase morphology of the impact fracture surface for PLA/PEBA-GMA/SiO_2_ specimen corresponded to the different points in Figure (a).

In the stress-whitened zone near the notch (Figure 7b), close to the stress initiation point, limited cavitation and occasional particle debonding were observed. According to the micromechanical model by Lazzeri and Bucknall,^43^ this indicated the initial response of the core–shell particles under high-strain-rate loading, where the elastomeric PEBA shell began to deform and absorb energy, while the rigid SiO_2_ cores acted as stress concentrators.

Moving toward the central region of the fracture surface (Figure 7c), the morphology became relatively smoother, with evident plastic flow lines. The strong interfacial adhesion provided by the GMA grafting ensures that the PEBA shell remains bonded to the matrix, acting as a bridge to transfer load. The PEBA shell undergone larger deformation, facilitating stress transfer to the PLA matrix. The presence of SiO_2_ cores promoted localized shear yielding of the surrounding matrix, leading to the initiation of plastic deformation.

In the hinge region (Figure 7d), massive shear yield and strong plastic deformation were dominant, characterized by fibrillar structures and pronounced ductile tearing. This reflected the full activation of the core–shell particles in inducing massive matrix plasticity. The PEBA shell not only served as a stress-transmitting medium but also cavitates and elongates, thereby relieving triaxial stress and enabling large-scale shear band formation in the PLA matrix.

Throughout the fracture process, the SiO_2_ cores—encapsulated by the PEBA elastomer—function as efficient stress concentrators. They helped convert localized stress concentration into widespread matrix yield, thereby enabling high energy dissipation via plastic deformation. This multi-zone, progressive toughening behavior underscored the role of the core–shell architecture in achieving a brittle-to-ductile transition in PLA, consistent with findings reported in other toughened PLA blend systems such as PLA/PBS/PBAT and PLA/EGMA/PA11.^26,34,38^

## 4 Conclusions

In this work, high-performance poly­(lactic acid) (PLA) composites with an excellent balance of stiffness and toughness were successfully fabricated by incorporating SiO_2_–PEBA core-shell particles via simple melt blending. Morphological characterizations (SEM and TEM) combined with spreading coefficient calculations have confirmed the formation of a thermodynamically favored, well-defined core-shell structure.

A systematic investigation revealed that the SiO_2_ core particle size and core-to-shell mass ratio are pivotal parameters governing the final performance. Severe agglomeration of small SiO_2_ particles (100 nm) compromised the dispersion and led to ineffective toughening. In contrast, the PLA composites incorporating 500 nm SiO_2_ at a core-shell ratio of 3:1 and a total modifier content of 20 wt % exhibited an optimal combination of properties, achieving a notched Izod impact strength of 33.9 kJ/m^2^—nearly 10 times that of neat PLA—while retaining 87% of the flexural modulus of pure PLA.

Melt rheology analysis revealed that the elastomeric shells formed a physical network, enhancing the melt elasticity. Conversely, increasing the rigid SiO_2_ core content effectively diluted this entanglement density, restoring liquid-like relaxation dynamics. This unique rheological behavior ensures that the composites maintain excellent processability.

The synergistic toughening mechanism is attributed to the cavitation-induced shear yielding process: the PEBA shell cavitates to relieve hydrostatic pressure, triggering massive matrix shear yielding, while the rigid SiO_2_ cores act as stress concentrators and maintain high stiffness. Furthermore, the interfacial chemical bonding provided by GMA grafting was crucial for stabilizing the morphology and ensuring an efficient stress transfer between the phases.

This work provides a scalable and effective strategy to resolve the inherent stiffness-toughness trade-off in PLA, expanding their potential for high-performance structural material applications.

## Supplementary Material



## Data Availability

The data in this study can be available from the corresponding author.

## Notes

Compositions of PLA/PEBA-GMA/SiO_2_ composites; SEM images of PLA/PEBA-GMA/SiO_2_ composites with 30 wt % modifier; Histograms of particle size distribution for 100 and 500 nm SiO_2_ systems; Rheological strain sweep results; Rheological time sweep results (PDF)
