# Supplementary material for: Core-Shell Structure Strategy to Prepare Super-tough Poly(lactic acid) Composites with Balanced Stiffness and Toughness
Source: Polym Sci Technol. 2026 Apr 14;2(7):505–14. doi: 10.1021/polymscitech.6c00012 (PMC13420098; doi:10.1021/polymscitech.6c00012)
Supplement: Supplementary file 1 [file ps6c00012_si_001.pdf]

Supporting Information

Core-shell structure strategy to prepare super-tough poly(lactic acid) composites with balanced stiffness and toughness

Wei Bao[1,2], Xiaodong Wang[1,2], Lei Li[,2], Jing Jin[2,3], Yanxiong Pan[1,2], Hongwen Liang[4], Xiangling Ji[1,2]*, Wei Jiang[1,2,3]*

[1] University of Science and Technology of China, Hefei 230026, People's Republic of China

[2] State Key Laboratory of Polymer Science and Technology, Changchun Institute of Applied Chemistry, Chinese Academy of Sciences, Changchun 130022, People's Republic of China

[3]State Key Laboratory of New Textile Materials and Advanced Processing Technologies and Key Laboratory of Textile Fiber and Products of Ministry of Education, College of Materials Science and Engineering, Wuhan Textile University, Wuhan 430200, People's Republic of China

[4]Hunan Petrochemical Co Ltd, Sinopec, Yueyang 414014, People's Republic of China

E-mail: xlji@ciac.ac.cn, weijiang@wtu.edu.cn

Table S1 Composition for PLA/PEBA-GMA/SiO_2 composites

Group		Modifier (PEBA-GMA) (%)		PLA (%)
		0		100
I		10		90
		15		85
		20		80
	SiO_2 size/ nm	SiO_2:PEBA-GMA	%	%
			5	95
		1:3	10	90
			15	85
			20	80
			30	70
	100		5	95
		1:2	10	90
	200		15	85
II			20	80
	300		30	70
			5	95
	500	1:1	10	90
			15	85
	800		20	80
			30	70
			5	95
		2:1	10	90
			15	85
			20	80
			30	70
			5	95
		3:1	10	90
			15	85
			20	80
			30	70

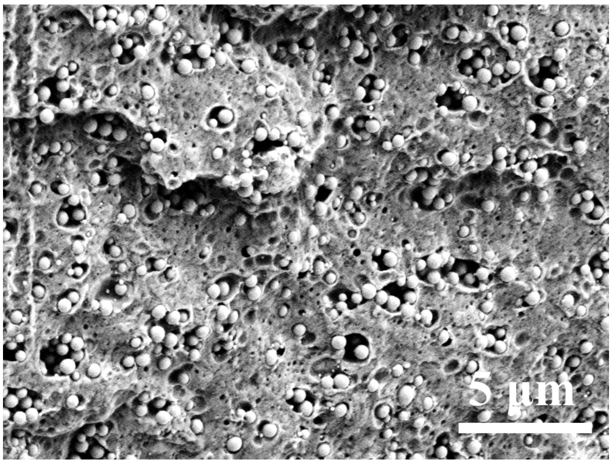

Figure S1. SEM images showing phase morphology for PLA/PEBA/SiO_2-500nm composites with 30 wt% SiO_2-PEBA core-shell particle sizes. The core-shell mass ratio is 3:1. The cryo-fracture surfaces were etched in n-butanol at 80 ℃ for 12 h to remove the PEBA.

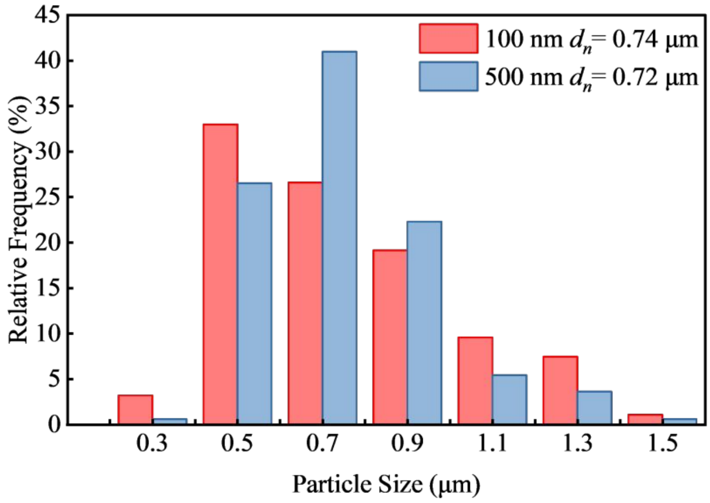

Figure S2. Histograms of particle size distribution for PLA/PEBA-GMA/SiO_2 composites. The PEBA-GMA-SiO_2 core-shell ratio is 1:1. The addition of core-shell content is 20 wt%.

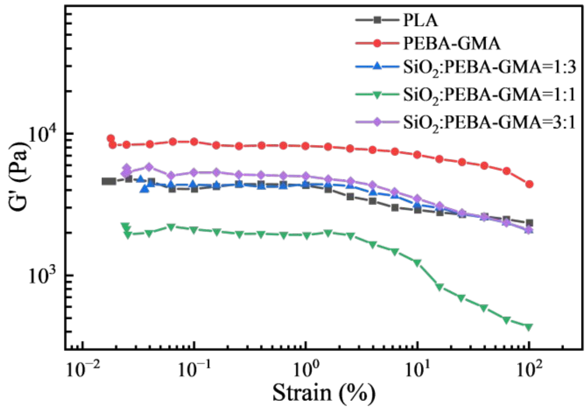

Figure S3. Rheological properties of PLA/PEBA-GMA/SiO_2 composites with different SiO_2:PEBA-GMA mass ratios as a function of strain.

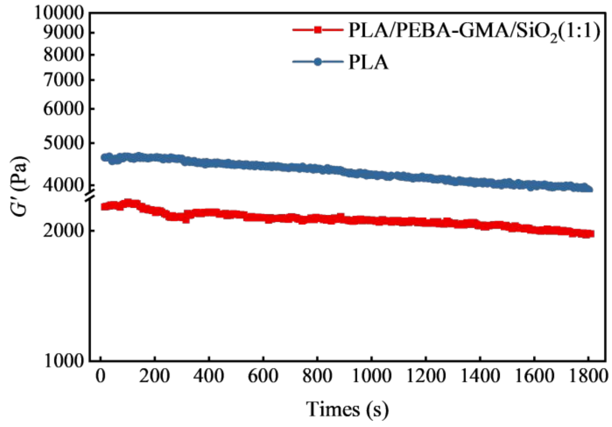

Figure S4. Storage modulus of neat PLA and PLA/PEBA-GMA/SiO_2-500nm composites as a function of time.
